# Impact of COVID-19 pandemic on primary care research in a resource-limited setting: a commentary

**DOI:** 10.11604/pamj.supp.2020.35.2.22724

**Published:** 2020-04-30

**Authors:** Godpower Chinedu Michael, Ibrahim Aliyu

**Affiliations:** 1 Department of Family Medicine, Aminu Kano Teaching Hospital, Kano, Nigeria; 2 Department of Paediatrics, Bayero University Kano, Aminu Kano Teaching Hospital, Kano, Nigeria

**Keywords:** COVID-19, pandemic, research, activities, resource-limited setting

## Abstract

The COVID-19 pandemic has impacted on several aspects of human existence including primary care research activities in resource-limited settings. Opportunities exist for initiating multi-disciplinary collaborative research teams that may examine current controversial areas of the disease such as prevention, diagnosis and treatment; experiences of stakeholders like COVID-19 survivors and frontline health workers; and individuals and community experiences during lockdowns. Challenges associated with initiating new studies and/or sustaining old ones and publication of research outcomes may need to be curtailed through alternative strategies and support from stakeholders.

## Commentary

COVID-19 is an infectious disease caused by the novel coronavirus, CoV-2 (SARS-CoV-2) [1]. The disease was declared a global pandemic by the World Health Organization (WHO) on 11 March 2020 [2]. As at 12p.m. ET, 20 April 2020 there were at least 14760 cases and 662 deaths in 46 African countries [3]. Globally, national responses towards curtailing the spread and mortality from COVID-19 have largely depended on the strength of existing healthcare systems, economic capabilities, perceived degree of affectation and politics. Though the effectiveness of these responses can hardly be evaluated now, the level of preparedness and response from most healthcare systems (including those of high-income countries) have been a subject of discuss by many commentators [4]. However, handwashing with running water and soap (or alcohol hand rubs), social distancing and lockdown of cities, states and countries (though of doubtful efficacy) [5] are common interventions globally employed. The disease and its interventions have also thrown up opportunities and challenges in almost every aspect of human existence. These challenges expectedly are more pronounced in Africa with the highest deposits of poor people [6]. One neglected area in resource-limited settings is the impact of the pandemic on research activities (especially in primary care).

COVID-19, as a new disease entity with controversies surroundings its prevention, diagnosis and treatment, offer opportunities for new studies. Firstly, areas such as determination of the utility of facemasks, point-of-care diagnostics, and optimization of personal protective equipment should be considered for research [7]. Some of these research ideas may appear daunting for primary care researchers in Africa; however, the formation of national and international multi-disciplinary collaborative research teams can reduce the challenge. In addition, studies on the unique experiences (including mental health) of COVID-19 survivors, patients who tested negative despite meeting case definitions and frontline healthcare providers also constitute opportunities for research at this time. Secondly, the imposition of lockdown in cities and countries in Africa makes studies on the unique experiences, behaviour and coping strategies of individuals and communities during the lockdown necessary. Some media reports suggest increasing incidences of domestic violence and legal suits for divorce since the imposition of lockdown in some high-income countries. This is also another area for research. However, studies in these domains will require multi-disciplinary research (that incudes sociologists, anthropologists, etc.). Lastly, the lockdown offers an important time to undertake research activities such as writing research proposals, manuscripts of completed studies and commence data collection for online or email surveys (depending on availability of resources).

Among the challenges to research activities in resource-limited primary care settings is how to start new studies. There are delays in obtaining ethical approvals from Institutional Review Boards; some boards are no longer sitting because of the need to enforce social distancing. E-meetings (e.g. with skype or zoom) can be used to surmount this challenge. Secondly, ongoing studies at the stage of data collection are also affected. The current reduction in patient turnout in medical facilities occasioned by movement restriction and restriction of number of primary care patients by some medical facilities to implement measures such as social distancing has its consequences on research activities. Collection of quantitative or qualitative data (via focus group discussions) may be affected. This may result in deviation from and modification of approved study protocols, some studies will require new ethical approvals, study duration may become prolonged and sometimes the study is outrightly suspended (e.g. studies in which temporal bias is anticipated). Excessive attrition could also occur in some studies (e.g. clinical trials) due to the lockdown effect and thus affecting outcome. Thirdly, the involvement of healthcare professionals (e.g. those in the frontline) reduces the availability of journal reviewers at this time; this eventually delays publication of submitted manuscripts. Finally, journal editors appear pressured to publish manuscripts concerning COVID-19 or other related diseases over non-COVID-19 related manuscripts currently. This is probably because COVID-19 manuscripts are likely to be more appealing to readers. However, journal editors should try and balance the choice of manuscripts for processing and publication to encourage researchers at this time. In conclusion, while disease outbreaks will never cease, adaptive measures are required to curtail its effects on primary care research activities. Researchers if possible, should see this period as an opportunity for new research frontiers and seek ingenious ways of circumventing challenges at this time. More studies are also needed to offer alternative strategies to navigate the various challenges to research activities in resource-limited settings during pandemics and similar times.

## Competing interests

The authors declare no competing interests.
